# Sporadic and MEN1-related gastrinoma and Zollinger–Ellison syndrome: differences in clinical characteristics and survival outcomes

**DOI:** 10.1007/s40618-022-01961-w

**Published:** 2022-11-27

**Authors:** S. Massironi, R. E. Rossi, A. Laffusa, C. Eller-Vainicher, F. Cavalcoli, A. Zilli, C. Ciafardini, V. Sciola, P. Invernizzi, M. Peracchi

**Affiliations:** 1 Division of Gastroenterology and Center for Autoimmune Liver Diseases, San Gerardo Hospital, Via Pergolesi 3, Monza, Italy; 2 Department of Medicine and Surgery, European Reference Network on Hepatological Diseases (ERN RARE LIVER), San Gerardo Hospital, University of Milano-Bicocca, Monza, Italy; 3 Gastroenterology and Endoscopy Unit, IRCCS Humanitas Research Hospital, Via Manzoni 56, Rozzano, 20089 Milan, Italy; 4 Endocrinology, Fondazione IRCCS Ca’ Granda Ospedale Policlinico di Milano, Milan, Italy; 5 Diagnostic and Therapeutic Endoscopy Unit, Fondazione IRCCS Istituto Nazionale Tumori, Milan, Italy; 6 Gastroenterology and Endoscopy, IRCCS Ospedale San Raffaele, Vita-Salute San Raffaele University, Milan, Italy; 7 Gastroenterology and Endoscopy, Fondazione IRCCS Ca’ Granda Ospedale Policlinico di Milano, Milan, Italy

**Keywords:** Zollinger–Ellison syndrome, Gastrinoma, Multiple endocrine neoplasia type 1, MEN-1, Neuroendocrine tumors, Neuroendocrine neoplasms

## Abstract

**Purpose:**

Gastrinoma with Zollinger–Ellison syndrome (ZES) may occur sporadically (Sp) or as part of the inherited syndrome of multiple endocrine neoplasia 1 (MEN-1). Data comparing Sp and MEN-1/ZES are scanty. We aimed to identify and compare their clinical features.

**Methods:**

Consecutive patients with ZES were evaluated between 1992 and 2020 among a monocentric Italian patient cohort.

**Results:**

Of 76 MEN-1 patients, 41 had gastroenteropancreatic neuroendocrine neoplasm (GEP-NEN), 18 of whom had ZES; of 320 Sp-GEP-NEN, 19 had Sp-ZES. MEN-1/ZES patients were younger (*p* = 0.035) and the primary MEN-1/ZES gastrinoma was smaller than Sp-ZES (*p* = 0.030). Liver metastases occurred in both groups, but only Sp-ZES developed extrahepatic metastases. 13 Sp-ZES and 8 MEN-1/ZES underwent surgery. 8 Sp-ZES and 7 MEN-1/ZES received somatostatin analogs (SSAs). Median overall survival (OS) was higher in MEN-1/ZES than in Sp-ZES (310 vs 168 months, *p* = 0.034). At univariate-logistic regression, age at diagnosis (*p* = 0.01, OR = 1.1), G3 grading (*p* = 0.003, OR = 21.3), Sp-ZES (*p* = 0.02, OR = 0.3) and presence of extrahepatic metastases (*p* = 0.001, OR = 7.2) showed a significant association with OS. At multivariate-COX-analysis, none of the variables resulted significantly related to OS. At univariate-logistic regression, age (*p* = 0.04, OR = 1.0), size (*p* = 0.039, OR = 1.0), G3 grade (*p* = 0.008, OR = 14.6) and extrahepatic metastases (*p* = 0.005, OR = 4.6) were independently associated with progression-free survival (PFS). In multivariate-COX-analysis, only extrahepatic metastases (*p* = 0.05, OR = 3.4) showed a significant association with PFS. Among SSAs-treated patients, MEN-1/ZES showed better PFS (*p* = 0.0227)**.** After surgery, the median PFS was 126 and 96 months in MEN-1 and Sp, respectively.

**Conclusion:**

MEN-1/ZES patients generally show better OS and PFS than Sp-ZES as well as better SSAs response.

## Introduction

Zollinger–Ellison syndrome (ZES) is a clinical manifestation associated with an underlying gastrinoma of the duodenum or pancreas, a neuroendocrine neoplasm (NEN) that can cause hypersecretion of gastric acid, typically leading to gastroesophageal reflux disease (GERD), recurrent peptic ulcers and chronic diarrhea due to hypersecretion of gastrin.

Gastrinoma is the most common functional duodenal NEN (dNEN) and the second most common functional pancreatic NEN (pNEN), after insulinoma. Gastrinoma usually occurs sporadically (Sp-ZES) and is diagnosed between the ages of 50 and 70 years with a male-to-female ratio of 1.5–2:1 [1], while 20–30% of patients develop ZES as part of a genetic syndrome known as multiple endocrine neoplasia type 1 (MEN-1) (MEN-1/ZES) [2].

MEN -1 is an autosomal dominant disorder with an incidence ranging from 16 to 38% in patients with gastrinomas [3] and characterized by the presence of parathyroid adenomas/hyperplasia (90%), gastroenteropancreatic (GEP)-NEN (30–70%), mostly a gastrinoma (40%), and pituitary adenomas (30–40%). In patients with MEN-1/ZES, the development of hypercalcemia secondary to hyperparathyroidism is one of the earliest signs. In these cases, diagnosis is often challenging due to several confounding factors, e.g., hyperparathyroidism associated with chronic atrophic gastritis and concomitant use of proton pump inhibitors (PPI) [4, 5]. In addition, correct radiological diagnosis and staging can be difficult in this particular situation because of the presence of multiple tumors smaller than 1 cm [6]. To get a definitive diagnosis, clinical suspicion should arise in the presence of concomitant symptoms related to ZES (i.e. chronic diarrhea, recurrent peptic ulcer and GERD) and primary hyperparathyroidism, especially in young patients; the genetic test for MEN-1 syndrome is generally indicated in a selected subset of patients, namely: (1) in patients with two or more MEN-1-associated primary endocrine tumors or hypercalcemia associated with a GEP-NEN; and (2) in patients who show MEN-1 related features and are first-degree relatives of a patient with a clinical diagnosis of MEN-1.

In patients with MEN-1/ZES, gastrinoma occurs on average 5 to 10 years earlier than in Sp forms, usually before the age of 50 [7]; it occurs mostly in the duodenum (85–100%) and less frequently in the pancreas (till 15%), with tumors that are often multiple, small (even < 0.5 cm) and associated with lymph node metastases in 40–60% of the cases [8].

However, there are aspects that have not been fully clarified in the two forms of the disease; in particular, it has not been clearly defined whether the clinical course is the same in the familial and Sp forms and whether surgery has the same indications in both subgroups. Also, in terms of pharmacological treatment, whereas the therapy to control the syndrome is univocal and based on PPIs, the treatment of the tumor itself with somatostatin analogs (SSAs) is controversial [9, 10]. Moreover, surgical treatment of patients with MEN-1/ZES remains uncertain, even though according to the European Neuroendocrine Tumor Society (ENETS) guidelines, surgical resection with curative aim should be performed whenever possible in all ZES patients, both Sp and MEN-1/ZES [11, 12]. In unresectable patients, treatment with SSAs might be a valid option to control tumor, but available data in ZES patients are scanty, although ENETS guidelines recommend the use of SSAs in advanced, well-differentiated GEP-NENs as an antiproliferative agent [13, 14].

Since data comparing sporadic and hereditary forms are few and scanty, the aim of this study was to identify and compare clinical features and survival outcomes in Sp and MEN-1/ZES forms.

## Materials and methods

Between January 1992 and December 2020, 37 consecutive patients with gastrinoma/ZES diagnosed and followed up at the Department of Gastroenterology and Digestive Endoscopy of Fondazione IRCCS Ca’ Granda Ospedale Policlinico, Milan (until 2019), and subsequently at the Department of Gastroenterology of San Gerardo Hospital, Monza, Italy, were evaluated. The study was approved by the local Institutional Review Board (Comitato Etico Milano Area 2, Italy) and was conducted in accordance with the principles of the Declaration of Helsinki (revision of Edinburgh, 2000).

Baseline investigations were performed in all patients, including conventional radiology, functional imaging, and blood tests, including fasting gastrin levels.

The diagnosis of ZES was mainly based on (a) elevated fasting gastrin levels associated with gastric hyperchlorhydria, with a gastric pH of < 2, and (b) pathology reports of gastrin immunohistochemistry-positive tumors detected by endoscopy [gastroscopy and ultrasound endoscopy (EUS)] or on surgical specimens. Gastrinoma diagnosis was considered certain if the circulating gastrin level was more than ten times the upper normal limit (ULN). In uncertain cases, i.e. gastrin levels < 10 ULN, a secretin stimulation test was performed after multidisciplinary consultation. Each tumor was retrospectively classified immunohistochemically according to the WHO (World Health Organization) 2010 classification based on the Ki-67 index [15] when available, and the disease was staged based on the presence/absence of nodal or distant metastases, according to the current ENETS TNM (Tumor/Node/Metastases) clinical staging [16].

Plasma gastrin levels were prospectively collected at diagnosis and during follow-up. Venous blood samples were collected in EDTA-containing tubes between 8:00 and 10:00 am after an overnight fast. Samples were centrifuged at 4 °C and the plasma was separated and stored at − 30 °C until assayed. Gastrin was measured with commercially available kits.

Conventional radiology including computed tomography (CT) and magnetic resonance imaging (MRI) was performed at the time of diagnosis along with endoscopy and somatostatin receptor imaging [scintigraphy, Octreoscan^®^ or Gallium-68 positron emission tomography (PET) scan] and repeated every six months for the first five years and annually thereafter. Nuclear imaging and endoscopy were performed annually or in each suspicious case.

Patients who underwent surgical intervention were evaluated by biochemical and imaging tests 2–4 weeks after surgery and periodically thereafter every 6–12 months [11].

For patients treated by either surgically or drug therapy, the ITMO (Italian trials in medical oncology) group criteria were used to evaluate symptomatic, biochemical (plasma gastrin), and objective (lesion size) response, and were classified as complete or partial response in case of a decrease ≥ 50%; as stable disease if the decrease was < 50% or in case of an increase < 25%, as progressive disease if there was an increase greater than 25%. Genetic testing for MEN-1 was performed when indicated, that is, in patients with hypercalcemia associated with a NEN or two or more primary MEN-1 associated endocrine tumors or in presence of a positive family history.

All clinical data on primary tumor location and size, grading and staging, presence of multiple tumors, local spread or distant metastases, type of treatment (surgery, SSAs), and long-term outcomes, including overall survival (OS) and progression-free survival (PFS), were prospectively collected and retrospectively analyzed.

All data for each patient were anonymized after collection, recorded, evaluated and analyzed.

### Statistical analysis

Results are presented as median and interquartile range or mean and standard deviation (SD) unless otherwise stated. Categorical variables were expressed as number (percentage). Data were analyzed using non-parametric tests: Mann–Whitney test was used for comparison between groups. All data were tested for distribution normality by the Kolmogorov-Smirnoff test. Fisher’s exact test was used for the comparison of percentages.

OS was calculated from the time of ZES diagnosis until patient death or the end of data collection. PFS was defined as the time interval between successful surgical or pharmacological treatment and disease progression or until the end of data collection**.** Survival curves were estimated using the Kaplan–Meier method calculating the 95% confidence interval for fractional survival at a given time point. The log-rank test was used to compare the survival curves between patient groups. The univariate and multivariate Cox regression models were used to analyze the possible association between the variables of interest (age, sex, size and location of the primary tumor, TNM staging, Ki-67 index, SSAs treatment, surgical treatment, and presence of MEN-1 syndrome) and the risk of death and progression. The best multivariate model was identified using a stepwise forward method (entry criterion: *p* < 0.05; removal criterion: *p* > 0.1). The estimated hazard ratios (HR), as derived from the Cox models, were reported along with the pertinent 95% confidence intervals (CIs). A *p *value < 0.05, two-sided, was considered statistically significant. Analyzes were performed using Graph Pad Prism version 6.00 software (GraphPad Software, San Diego, California, USA) and MedCalc version 17.9.5 (MedCalc Software bvba, Ostend, Belgium).

## Results

A total of 76 MEN-1 and 320 Sp-GEP-NEN patients were evaluated. Forty-one MEN-1 patients were diagnosed with GEP-NEN, of which 20 were non-functioning pNENs and 21 were functioning NENs. Among functioning NENs, three patients had insulinomas and 18 had gastrinomas with clinical manifestation of ZES. All the patients with MEN-1 syndrome were genetically diagnosed, except for one patient who had clinical and familiar diagnostic criteria. Primary hyperparathyroidism was present in all the MEN-1/ZES patients, while a pituitary tumor was found in five of them. Among the Sp-GEP-NEN patients, 19 were diagnosed with Sp-ZES. A total of 37 ZES patients were studied and followed up for a median of 152 months [interquartile range (IQR) 46.5–200 months], with a median follow-up of 174 months (IQR 92.2–228) in MEN-1/ZES patients and 108 months (IQR 45–168) in Sp-ZES patients.

Among the Sp-ZES, 17 (89.4%) patients had peptic ulcer disease at the time of diagnosis, of whom one had concomitant mild diarrhea and one had severe esophagitis (Los Angeles C) with a major acute bleeding event, while two (10.6%) were paucisymptomatic, having already been treated with PPIs. In the MEN-1/ZES group, 15 patients (83.3%) had peptic ulcer disease at the time of diagnosis, of which two patients had major bleeding, two patients had esophagitis (Los Angeles C), and three patients had diarrhea, while three patients (16.7%) were paucisymptomatic due to ongoing PPI treatment. Clinical manifestation at the time of diagnosis was not related to patient outcome.

At the time of diagnosis, a total of 20 patients underwent CT imaging [11 Sp-ZES (57.9%) and 9 MEN-1/ZES (50%)], while 19 patients underwent MRI [9 patients with Sp-ZES (47.4%) and 10 patients with MEN-1/ZES (55.5%)]; two patients underwent both TC and MRI. Gallium-68 PET was performed in six Sp-ZES (31.6%) and three MEN-1/ZES patients (16.7%), while Octreoscan^®^ was performed in 12 Sp-ZES (63.2%) and 13 MEN -1/ZES patients (72.2%), somatostatin receptors functional imaging was not available in three patients diagnosed before 1995. Gallium-68 PET missed one patient with a positivity rate of 87.5%, whereas Octreoscan^®^ missed six patients with a positivity rate of 76%.

After diagnosis, all patients received appropriate PPI treatment with a good clinical response. The characteristics of the patients are shown in Table 1.

**Table 1:** Baseline characteristics of the patients divided by MEN-1/ZES and Sp-ZES

Characteristics	MEN-1/ZES patients (*N* = 18)	Sp-ZES patients (*N* = 19)	*p*
Age (years), mean (SD)	43.7 (± 12.3)	53.5 (± 14.7)	0.035
Female, *n* (%)	12 (66.6)	9 (47.3)	ns
Follow-up (months), median (IQR)	174.0 (92.2–228.0)	108.0 (45.0–168.0)	ns
Size of primary gastrinoma(mm), median (IQR)	13 (9–15)	18 (15–28)	0.03
Site of primary Duodenum, *n* (%) Pancreas, *n* (%) Liver, *n* (%) Gastric, *n* (%) Unknown, *n* (%)	10 (55.5) 5 (27.7) 1 (5.5) 0 (0) 2 (11.1)	7 (36.8) 5 (26.3) 1 (5.3) 1 (5.3) 5 (26.3)	ns
Liver metastases, *n* (%)	10 (55.5)	10 (52.6)	ns
Extra-hepatic metastases, *n* (%)	0 (0)	6 (31.6)	0.0197
Others NEN, *n* (%)	8 (44.4)	2 (10.5)	0.02
Other non-neuroendocrine neoplasms, *n* (%)	2 (11.1)	2 (10.5)	ns
Grading G1 G2 G3	11 (61.1) 3 (6.6) 0°	9 (47.4) 6 (31.6) 2° (10.5)	ns
TNM staging Stage I Stage I Stage III Stage IV	3 (16.6) 4 (22.3) 3 (16.6) 8 (44.5)	6 (31.6) 3 (15.8) 1 (5.2) 9 (47.4)	ns
Surgically treated patients, *n* (%)	8 (44.4)	13 (68.4)	ns
Somatostatin analogs treated, *n* (%)	7 (38.8)	8 (42.1)	ns
Gastrin levels (pg/mL) at diagnosis, median (IQR)	374.0 (215.8–631.5)	874.0 (464.0–1591.0)	0.012
Dead/alive, *n*	4/14	11/8	0.048

Not available for 4 MEN-1/ZES patients, and 2 Sp-ZES patients

MEN-1/ZES patients were younger than Sp-ZES (mean age 43.7 ± SD 12.3 vs 53.5 ± SD 14.7 years, *p* = 0.035), and primary gastrinoma was smaller in MEN-1/ZES compared to Sp-ZES (median13 vs 18 mm, *p* = 0.03). Primary dNEN was found in ten of 18 MEN-1/ZES patients and seven of 19 patients with Sp-ZES (55.5% and 36.8%, respectively, *p* = ns). The other patients with MEN-1/ZES had pancreatic (5/18), hepatic (1/18) and unknown primary localization (2/18). By contrast, the other patients with Sp-ZES had pancreatic (5/19), hepatic (1/19), gastric (1/19) and unknown primary localization (5/19). Gastrin plasma levels at diagnosis were higher in Sp-ZES than in MEN-1/ZES (median 874 vs 374 pg/ml, *p* = 0.012).

Liver metastases (either synchronous or metachronous) were found in ten (synchronous in eight and metachronous in two) Sp-ZES patients and in ten (synchronous in five and metachronous in five) MEN-1/ZES patients, respectively. Only the Sp-ZES patients developed extrahepatic metastases (6 versus 0, *p* = 0.019).

Two of 19 patients (10.5%) with Sp-ZES were found to have additional NENs at diagnosis or during follow-up, both gastric carcinoids. Eight of 18 patients (44.4%) with MEN-1/ZES had additional NENs, three non-functioning pNENs, two gastric carcinoids, two lung carcinoids, and one thymic carcinoid. Four of the 18 MEN-1/ZES also had a prolactinoma and one had a non-functioning pituitary adenoma.

Non-neuroendocrine tumors were observed during follow-up in two patients with MEN-1/ZES [one with gastrointestinal stromal tumor (GIST) and one with breast cancer] and in two Sp-ZES patients (one with prostate and one with breast cancer).

Surgery was performed in 13 Sp-ZES and eight MEN-1/ZES. Of the 13 Sp-ZES, 11 underwent duodenal-pancreatic (DP) surgery (one Whipple, two distal pancreatectomies, four pancreatic enucleations, four duodenotomies), one liver resection, and one gastrectomy. Six of 13 patients (46%) showed metastatic lymph nodes and seven patients had liver metastases (53%). Seven of the eight MEN-1/ZES patients (87%)underwent DP surgery (two distal pancreatectomies and five duodenotomies) and one liver resection; six (75%) had positive lymph nodes at the time of surgery and four (50%) showed liver metastases. At the initial postoperative follow-up (2–4 weeks), surgery resulted curative in all the patients with MEN-1/ZES and in 10/13 with Sp-ZES. However, at subsequent long-term follow-up, recurrences occurred in four Sp-ZES and four MEN-1/ZES, at 12–288 and 12–144 months, respectively. Among surgically treated patients, the PFS of the MEN-1/ZES patients was not statistically different from that of the Sp-ZES group, although a trend toward better PFS was observed for the MEN-1/ZES patients in the entire cohort (median PFS of 96 months for Sp-ZES and 126 months for MEN -1/ZES patients).

A total of 15 patients received SSAs (either octreotide 28 mg or lanreotide 120 mg every 28 days), eight of whom were in the Sp-ZES group and seven in the MEN-1/ZES group. Of the eight Sp-ZES patients, seven had liver metastases and one had liver and extrahepatic metastases. Of the seven MEN-1/ZES patients, six had liver metastases and two had multifocal pNEN**,** which was treated surgically in only one case. Two patients with Sp-ZES had a partial objective response while four Sp/ZES and six MEN-1/ZES showed stable disease. Disease progression occurred in two Sp-ZES and one MEN-1/ZES, and recurrence occurred in five Sp-ZES after 24–120 months (median 39).

During SSAs therapy, one MEN-1/ZES patient developed a lung carcinoid and another developed an esophageal GIST.

**Fig. 1:**
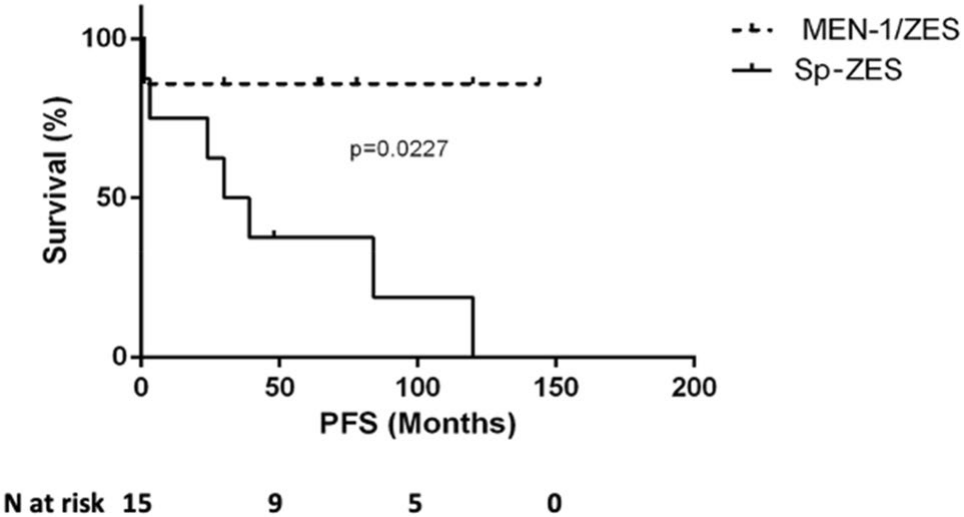
Progression-free survival of patients treated with somatostatin analogs in both MEN-1/ZES patients (not reached) and Sp-ZES (median 34.5 months, *p* = 0.0227 at log-rank-test)

With pharmacological treatment, MEN-1/ZES patients showed a better PFS than Sp-ZES (median PFS not reached vs 34.5 months, *p* = 0.0227 by log-rank-test) (Fig. 1).

**Fig. 2:**
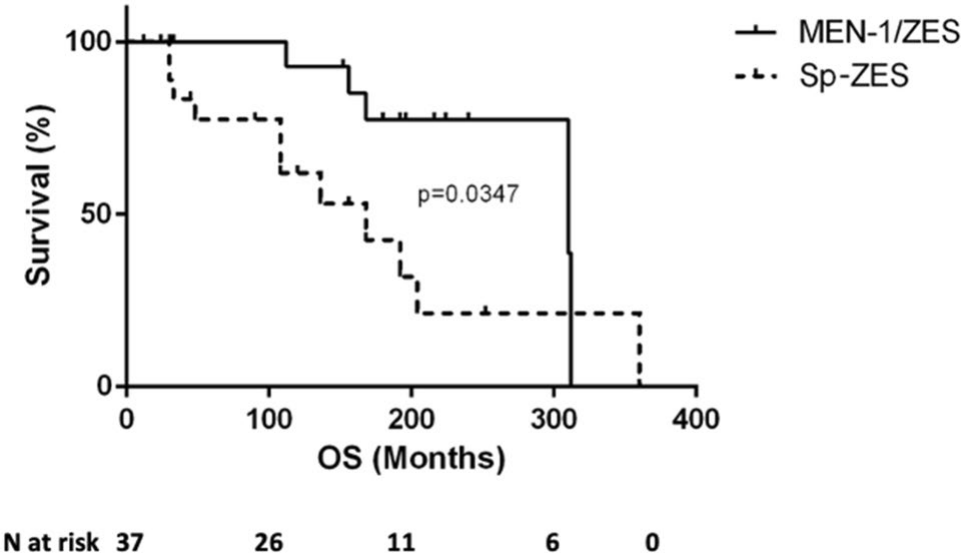
Median overall survival (OS) in MEN-1/ZES patients compared with Sp-ZES (310 vs 168 months, *p* = 0.0347 at log-rank-test)

The median OS (mOS) of the entire cohort was 204 months, and it was higher in MEN-1/ZES than in Sp-ZES (310 vs 168 months, *p* = 0.0347) (Fig. 2).

At univariate-logistic regression, age at ZES diagnosis [*p* = 0.01, odds ratio (OR) 1.05, CI 1.0–1.1], G3 grading (*p* = 0.003, OR 21.3, CI 2.8–162.0), MEN-1/ZES (*p* = 0.02, OR 0.3, CI 0.1–0.9) and extrahepatic metastases (*p* = 0.001, OR 7.2, CI 2.2–23.4) were significantly related to OS (Table 2), while TNM, gender, gastrin level, location and size of primary tumor did not matter.

**Table 2:** Univariate and multivariate analyses of overall survival (OS)

Covariate	Univariate *p *value	OR (95% CI)	Multivariate *p *value	OR (95% CI)
Age	0.01*	1.05 (1.012–1.11)	0.49	1.01 (0.96‒1.07)
Grading (G3)	0.003*	21.32 (2.80–162.00)	0.16	5.73 (0.49‒66.58)
MEN-1	0.02*	0.27 (0.08–0.86)	0.15	0.34 (0.07–1.50)
Extra-hepatic metastases	0.001*	7.19 (2.20–23.45)	0.23	2.40 (0.56–10.34)
Gender	0.22	2.01 (0.63–6.38)	–	–
TNM	0.57	0.53 (0.14–2.01)	–	–
Size	0.06	1.04 (0.99–1.09)	–	–
Surgery	0.46	1.51 (0.50–4.48)	–	–
Site	0.36	3.03 (0.72–12.77)	–	–
SSAs	0.32	1.73 (0.58–5.19)	–	–
Circulating gastrin	0.35	0.99 (0.99–1.00)	–	–

*p < 0.05

**Fig. 3:**
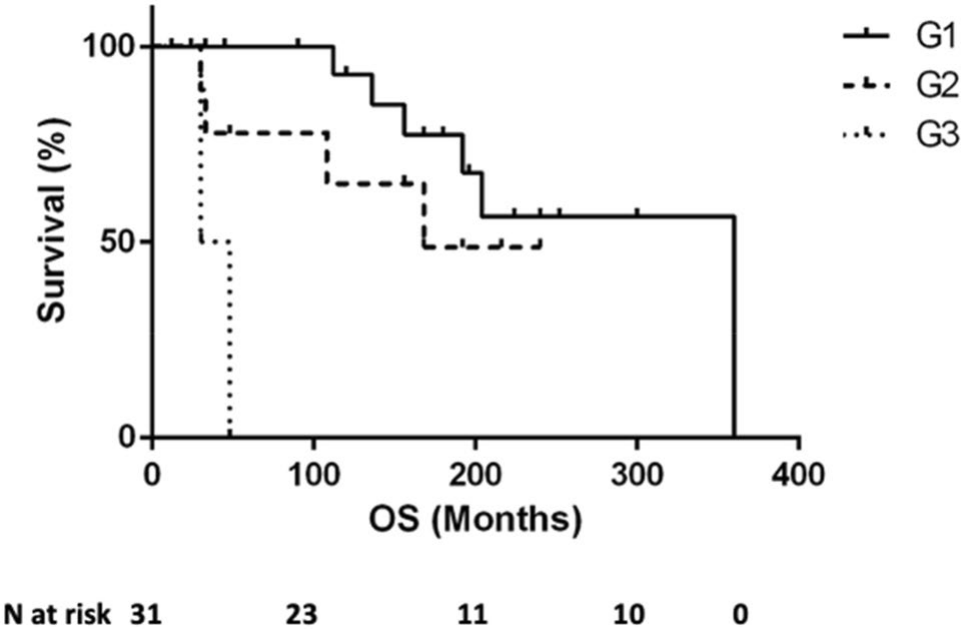
Overall survival in the entire study population, according to grading

The grading affected the mOS, which was 310 months in G1 patients, 168 months in G2 patients, and 39 months in G3 patients (*p* = 0.003 by log-rank-test) (Fig. 3). Moreover, even though TNM did not affect OS, the presence of extrahepatic metastases, which occurred only in Sp-ZES, resulted in a significant shortening of OS. However, among patients with liver metastases, OS was better in the MEN-1/ZES group (mOS Sp-ZES 96 months vs MEN-1/ZES not reached, *p* = 0.0069 by log-rank-test).

Gastrin levels at diagnosis did not correlate with OS, although patients who died due to ZES progression had significantly elevated circulating gastrin levels before death (median 16,815 pg/ml, IQR 695.5–59,375) compared to median at diagnosis (1090 pg/ml, IQR 625–1835, *p* = 0.0098).

In multivariate regression analysis, none of the variables was independently associated with OS (Table 2).

Regarding PFS, at univariate-logistic regression, age (*p* = 0.04, OR 1.0, CI 1.0–1.1), size (*p* = 0.039, OR 1.0, CI 1.0–1.1), G3 grade (*p* = 0.009, OR 14.6, CI 2.0–107.6) and the presence of extrahepatic metastases (*p* = 0.005, OR 4.6, CI 1.6–13.8) were independently associated with PFS (Table 3). At multivariate regression analysis, only the presence of extrahepatic metastases (*p* = 0.05, OR 3.4, CI 0.99–11.6) resulted independently associated with PFS (Table 3).

**Table 3:** Univariate and multivariate analyses of progression-free survival (PFS)

Covariate	Univariate *p *value	OR (95% CI)	Multivariate *p *value	OR (95% CI)
Age	0.04*	1.03 (1.00–1.06)	0.16	1.02 (0.99–1.05)
Grading (G3)	0.009*	14.60 (1.98–107.58)	0.21	3.95 (0.45–34.36)
MEN-1	0.48	1.34 (0.59–3.04)	–	–
Extra-hepatic metastases	0.005*	4.64 (1.56–13.77)	0.05*	3.4 (0.99–11.6)
Gender	0.08	2.08 (0.90–4.78)	–	–
TNM	0.15	1.83 (0.79–4.18)	–	–
Size	0.039*	1.03 (1.00–1.06)	0.26	1.01 (0.98–1.04)
Surgery	0.43	0.68 (0.27–1.74)	–	–
Site	0.14	0.84 (0.36–1.94)	–	–
SSAs	0.06	2.32 (0.97–5.55)	–	–
Circulating gastrin	0.13	0.99 (0.99–1.00)	–	–

*p < 0.05

At the end of the study, eight Sp-ZES (42%) and 14 MEN-1/ZES (78%) were alive (*p* = 0.0448 Fisher-test), 9/19 (47%) and 3/18 (17%) patients died of ZES in Sp-ZES and MEN-1/ZES, respectively. In the MEN-1/ZES group, another patient died from malignant thymic carcinoma. In the Sp-ZES group, besides the nine ZES-related deaths, two other patients died because of other non-endocrine malignancies. Age at death was not significantly different in the four MEN-1/ZES patients (median 61.5 years, IQR 52.7–67.2) and the 11 Sp-ZES (median 71 years, IQR 51–80).

## Discussion

ZES related to the presence of an underlying gastrinoma may occur sporadically or be part of the MEN-1 syndrome. To date, there are few studies that have specifically addressed these two groups, and there is no clear comparison between them. Therefore, the present work aims to compare these two cohorts of patients to understand potential differences that could impact their clinical management.

The epidemiological data of our study confirm the observations made previously that MEN-1/ZES patients are diagnosed at a younger age than Sp-ZES [7]. Furthermore, we observed that the primary tumor was smaller in MEN-1/ZES compared to Sp-ZES (median 13 vs 18 mm), as previously described in MEN-1 patients with small (often < 1 cm) and multiple tumors [6]. Although this could be related to the different biological behavior between MEN-1 neoplasms and Sp forms, it could also be the result of earlier diagnosis due to ongoing screening protocols in established MEN-1 patients. In addition, they may present other symptoms due to associated endocrinopathies, which require early medical attention. Regarding the localization of gastrinoma, we did not find significant differences in primary localization between MEN-1/ZES and Sp-ZES: in both groups, the duodenal wall was the most common site of origin, accounting for 55.6% of MEN-1/ZES cases and 36.8% of Sp-ZES cases, in contrast to previous studies indicating that the MEN-1/ZES primary lesion is localized in the duodenal wall in up to 85–100% of cases and less frequently in the pancreas [8]. Interestingly, Sp-ZES had a higher prevalence of gastrinomas of unknown origin (26.3% versus 11.1%). Again, as previously described, we observed GEP-NEN occurrence in 54% of MEN-1 patients, with ZES accounting for 44% of GEP-NEN cases in this setting [17]. In our cohort of patients, among the MEN-1/ZES patients, we observed the coexistence in two cases of a gastrinoma and another second primary non-endocrine tumor; specifically, we observed a GIST in one case, already reported in the literature [18], and breast cancer in another case, which has also already been described [7].

As concerned Sp-ZES, a second primary tumor was observed in two patients (one prostate and one breast cancer), both associations already described [19].

According to our findings, MEN-1/ZES patients showed better OS and PFS compared to Sp-ZES patients. This finding is of interest, also considering that we did not observe a different age at death in MEN-1/ZES and Sp-ZES patients, despite reports in the literature suggesting that MEN-1 patients die at a younger age than their Sp counterparts [17].

Although in our study both Sp and MEN-1 gastrinomas have comparable metastatic potential in long-term follow-up (52.1% and 56.2% of cases, respectively), only the Sp forms developed extrahepatic metastases and had a higher rate of liver involvement at diagnosis (8 of 19 cases, 42.1%) than MEN-1/ZES cases (5 of 18, 27.8%). This might be partially responsible for the worse OS and PFS of Sp tumors, along with the older age and larger size of the primary tumor. This observation could reinforce the idea of early diagnosis of gastrinoma in MEN-1 patients, also considering that this difference tends to level up over time, but may be due to different biologic behavior of the neoplasms in the two forms and it can be postulated that Sp-ZES may show different biological pathways in advanced disease conferring an increased ability to develop extrahepatic metastases.

As regards prognostic factors, data from our study suggest that patients with gastrinoma share similar prognostic factors to other NENs: for instance, advanced age at diagnosis has been demonstrated to be a risk factor for worse survival in all NENs [20, 21]; this might be due to the presence of co-morbidities and reduced tolerance to treatments, which could limit the number of therapeutic options. Histological grade has already been confirmed as a key prognostic factor for GEP-NENs, and in our study we indeed confirmed that higher histologic grades are relevant prognostic factors in both Sp and MEN-1/ZES patients [15]. Moreover, according to previously published data in GEP-NENs, the presence of extrahepatic metastases is an important prognostic factor for ZES patients even in the presence of hepatic involvement [21, 22].

In terms of treatment, the role of surgery in MEN-1 patients is still a matter of debate, also considering, together with some technical issues, that these tumors are often multiple, have lymph node metastases and are characterized by a high risk of recurrence; however, available guidelines generally suggest surgical removal of the primary tumor whenever possible also in the presence of MEN-1 syndrome [11, 12, 23]. Herein, surgery was performed in 13 Sp-ZES and eight MEN-1/ZES, which is partially in line with data from the literature [24]**.** Surgery was curative in all the MEN-1/ZES and in 77% (10/13) of Sp-ZES patients, although in the long-term follow-up recurrences occurred in four MEN-1/ZES and four Sp patients, with a median PFS of 126 and 96 months, respectively. These findings are relevant since patients with MEN-1/ZES often do not undergo surgery because the clinical syndrome is well controlled by PPI therapy.

All the patients received PPIs as per current guidelines [11, 12]. Furthermore, 15 patients received SSAs of whom eight (seven with liver metastases) were in the Sp-ZES group and seven (six with liver metastases) in the MEN-1/ZES one; among these, MEN-1 patients showed a better response to SSAs, thus confirming their antiproliferative effect in the treatment of metastatic gastrinomas. Indeed, the high expression of somatostatin receptors (SSRs) in gastrinomas makes them highly responsive to SSAs and supports the use of such drugs to counteract the tumor growth in patients not amenable to surgery [1, 14]. However, only a few data, mainly based on case reports or small retrospective series, support their use in advanced gastrinomas and no data are available in the literature on differences in response between Sp and hereditary forms, so it is difficult to quantify their ability to control tumor growth and disease progression. However, the significance of the better response to SSAs in MEN-1/ZES patients, which we observed in our cohort, needs to be further confirmed; even if we might speculate that MEN1- patients show a better response due to overexpression of SSRs, we have also to consider that these patients are generally diagnosed at an earlier stage, show a smaller tumor burden and exhibit less aggressive behavior, as confirmed by the absence of extrahepatic metastases in the MEN-1 group; anyway, further studies are needed to draw more solid conclusions.

At the end of the study, a significant percentage of patients died from ZES in both groups. This could be related to a diagnostic delay, which is known to be usually longer than 5 years [25]: diagnosis is often made when the disease is too advanced for a surgical approach, and this could be responsible for the poor prognosis. The younger age at diagnosis in MEN-1/ZES could be a reason for differences in the outcome found in MEN-1 and Sp-ZES, however it is important to note that in our study age at diagnosis was not independently associated with either OS or PFS, as already described [26].

Our study has some limitations, including the retrospective nature and the long-time span (29 years) which might be accountable for the differences over the years in the available diagnostic and therapeutic tools. On the other hand, for the same reason, we can derive valuable information from this series reflecting the real-life clinical practice at a tertiary referral center for neuroendocrine tumors; furthermore, the monocentric nature of the study ruled out any risk of heterogeneity in the management of these patients.

In summary, MEN-1/ZES patients are generally younger, have a smaller primary tumor, do not present extrahepatic disease and show a better OS and PFS. It is important to keep in mind that ZES, whether sporadic or hereditary, is a malignancy that requires a prompt diagnosis and a subsequent treatment based on surgery, when possible, with curative intent to remove the primary tumor, and on PPIs for symptoms control. Of note, hereditary forms seem to respond better to treatment with SSAs, which might suggest starting them in early phases of the disease in patients with MEN-1/ZES, even if the underlying mechanisms for their antiproliferative effect are still unclear and further prospective studies are needed.

## Abbreviations

ZES: Zollinger–Ellison syndrome
NEN: Neuroendocrine neoplasm
GERD: Gastroesophageal reflux disease
dNEN: Duodenal neuroendocrine neoplasm
pNEN: Pancreatic neuroendocrine neoplasm
Sp: Sporadic
MEN-1: Multiple endocrine neoplasia type 1
GEP: Gastro-entero-pancreatic
PPIs: Proton pump inhibitors
SSAs: Somatostatin analogs
EUS: Ultrasound endoscopy
ULN: Upper limit of normal
CT: Computed tomography
MRI: Magnetic resonance imaging
PET: Positron emission tomography
OS: Overall survival
PFS: Progression-free survival
SD: Standard variation
HR: Hazard ratio
CIs: Confidence intervals
GIST: Gastrointestinal stromal tumor
DP: Duodenal-pancreatic
mOS: Median overall survival
SSRs: Somatostatin receptors
